# Narrative review: natural scaffolds used in vital pulp therapy

**DOI:** 10.5114/bta/225660

**Published:** 2026-08-04

**Authors:** Julia Grzegorczyk, Julia Kijowska, Wiktoria Jabłońska-Trzosek, Dominika Kołtun, Bartłomiej Ziomko

**Affiliations:** 1 Private Practice, Lublin, Poland; 2 Medical University of Lublin, Poland; 3 Private Practice, Radom, Poland; 4 Private Practice, Zamość, Poland

**Keywords:** vital pulp therapy, natural scaffolds, regenerative endodontics, dental pulp regeneration, pulp capping

## Abstract

Vital pulp therapy (VPT) is a modern approach to conservative treatment aimed at preserving and regenerating vital dental pulp. Its success largely depends on biomaterial scaffolds that support cell migration, differentiation, and proliferation, as well as angiogenesis and dentin regeneration. An electronic database search of PubMed, Scopus, and Google Scholar databases was conducted for publications from 2015 to 2025 using the following key words: “vital pulp therapy,” “natural scaffolds,” “regenerative endodontics,” “dental pulp regeneration,” and “pulp capping.” This paper reviews current research on select natural biomaterials used in VPT, including methacry-lated gelatin, chitosan, alginate, platelet-rich fibrin, and demineralized dentin matrix. Their biological, mechanical, and antibacterial properties are discussed, alongside their potential in regenerative endodontics. Special attention is given to their biocompatibility, modifiability, and capacity for controlled bioactive agent delivery. The paper also highlights the clinical limitations of these materials, such as a lack of standardization, high costs, and technical challenges. Despite promising *in vitro* and *in vivo* results, most of these biomaterials remain in the preclinical research phase. Consequently, the need for additional clinical trials and strategies that enable their routine clinical use is emphasized.

## Introduction

The dental pulp is vital for maintaining tooth vitality, supplying essential nutrients, and responding to harmful stimuli. Its primary functions include dentinogenesis via peripheral odontoblasts, sensory perception, protective functions through the formation of secondary and tertiary dentin, and immune responses during inflammation and tissue regeneration. Preserving pulp vitality is the cornerstone of conservative dentistry and essential to oral health. Regular dental checkups and, consequently, early caries diagnosis allow for timely implementation of vital pulp therapy (VPT).

VPT includes a range of procedures (Table 1), which preserve living tissues and the natural defense mechanisms of the tooth (Desai et al. 2025).

**Table 1:** Procedures used in vital pulp therapy (VPT)

VPT procedure	Description
Indirect pulp capping	Removal of the infected dentin while leaving a thin layer over the pulp, followed by the application of a biomaterial (e.g., mineral trioxide aggregate [MTA], Biodentine)
Direct pulp capping	Direct application of the biomaterial onto a pinpoint pulp exposure, which may be caused by accidental injury during caries removal
Partial pulpotomy (Cvek)	Removal of a small portion (2–3 mm) of the pulp chamber tissue while leaving deeper layers intact and covering the exposed area with a biological material
Full pulpotomy	Complete removal of pulp from the pulp chamber while leaving the vital pulp within root canals

The success of VPT largely depends on the material chosen, the degree of pulp inflammation, the patient’s age, and the extent of tissue damage (Desai et al. 2025). The materials used in VPT must be biocompatible, seal effectively, stimulate reparative dentin formation, and resist moisture. Additionally, they must possess antibacterial properties and promote tissue regeneration (Tong et al. 2022). Mineral trioxide aggregate (MTA) and Biodentine remain the gold standards; they are highly effective at stimulating reparative dentin formation due to their excellent biocompatibility and ability to form dentin bridges (Kunert and Lukomska-Szymanska 2020). In recent years, three-dimensional (3D) scaffolds have gained attention in regenerative therapy. These structures support cell migration, differentiation, and angiogenesis, enabling comprehensive regeneration of damaged pulp and dentin (Tong et al. 2022). Alongside stem cells and signaling molecules that guide tissue repair, scaffolds constitute a key component of the tissue engineering (TE) triad – which integrates biomedical engineering, materials science, and applied cell biology (Rajasekar et al. 2025). The main goal of TE is to restore or replace damaged organ functions. In this context, scaffolds serve as 3D supports for cells, signaling molecules, or drugs, providing an extracellular matrix that facilitates their transport and proper function without compromising material properties.

A wide range of biomaterials is available and can be classified by their origin as either natural or synthetic (Sequeira et al. 2024). Synthetic scaffolds, including polylactic acid, bioactive ceramic hydroxyapatite, and tricalcium phosphate, offer more predictable properties, mass production capabilities, and cost-effectiveness than natural scaffolds. However, their hydrophobic structure limits their bioactivity (Sequeira et al. 2024). In contrast, natural scaffolds contain bioactive molecules, growth factors, and signaling compounds that stimulate the differentiation of progenitor cells, which is essential for effective tissue regeneration (Sequeira et al. 2024). These include materials such as methacrylated gelatin (GelMA), chitosan, alginate, platelet-rich fibrin (PRF), and demineralized dentin matrix (DDM). The present review focuses on these natural scaffolds because recent research indicates they yield superior cell viability and dental pulp-like tissue formation compared to synthetic alternatives (Shobana et al. 2022).

Figure 1 shows the classification of injectable hydrogels based on their origin. Special attention is given to natural hydrogels, as they constitute a significant part of this study (Islam et al. 2025).

**Figure 1:**
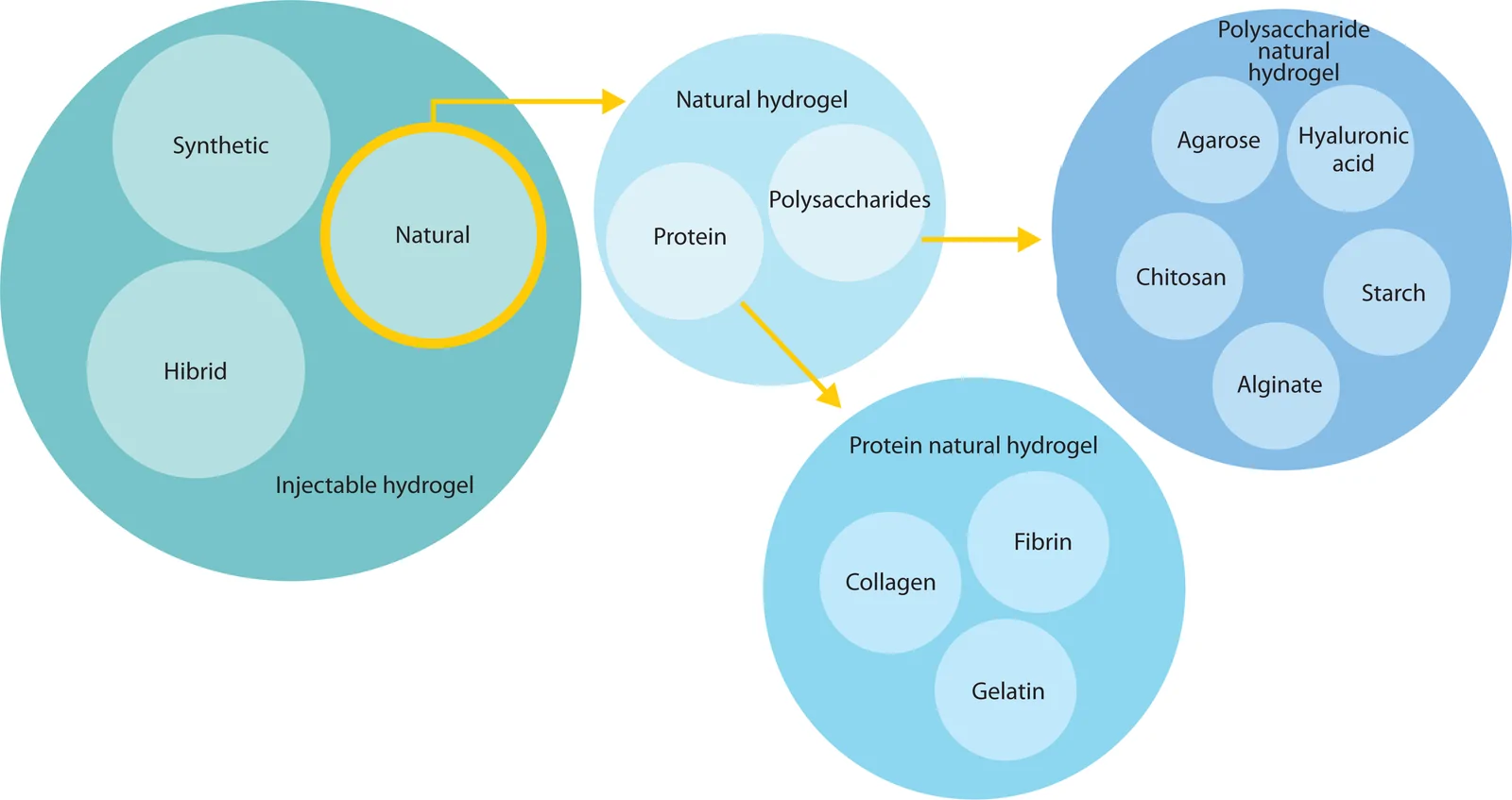
Classification of injectable hydrogels based on their origin. Special attention has been given to natural hydrogels, which constitute a significant part of this study

The aim of the study is to comprehensively characterize innovative biomaterial scaffolds for VPT. Their biological, mechanical, and antibacterial properties – which are critical for the protection and regeneration of the vital pulp – are evaluated and compared. Particular emphasis is placed on how effectively these biomaterials stimulate the proliferation, migration, and differentiation of dental pulp stem cells (DPSCs).

## Materials and methods

A narrative literature review was conducted across PubMed, Scopus, and Google Scholar databases for English-language publications from 2015 to 2025. The search was carried out using the keywords “vital pulp therapy,” “natural scaffolds,” “regenerative endodontics,” “dental pulp regeneration,” and “pulp capping.” Articles that evaluated natural scaffolds used in biological pulp treatment were selected based on their thematic relevance. Priority was given to original research and preclinical studies detailing the composition, antibacterial properties, mechanisms of action, modifications, and regenerative potential of natural scaffold materials used in VPT. Due to the narrative nature of this review, no formal systematic protocol was applied. The selected literature informed the subsequent analysis of biomaterials, including their properties, possible modifications, and future development perspectives.

## Results

### Collagen, gelatin, and methacrylated gelatin

Gelatin is a derivative of collagen formed through partial hydrolysis. Both substances exhibit weak mechanical properties and rapid degradation, requiring modification for effective clinical application (Lv et al. 2023). One such modification is methacrylation. GelMA is a biocompatible, biodegradable, light-curable hydrogel (Sequeira et al. 2024) synthesized by reacting gelatin side chains containing amines with methacrylamide and methacrylate groups (Swanson et al. 2023).

Numerous reports indicate that GelMA serves as an effective carrier for drugs and cells. GelMA has been successfully modified with chlorhexidine-loaded nanotubes (Ribeiro et al. 2020a). This material demonstrates strong anti-inflammatory potential, which is clinically relevant since periapical inflammation involves the release of matrix metalloproteinases (MMP-1, MMP-2, MMP-8, and MMP-9) that degrade the extracellular matrix (Cabral-Pacheco et al. 2020). MMP activity degrades GelMA, triggering the release of chlorhexidine, a well-documented antibacterial agent (Khoury et al. 2024). This modification creates an “on-demand” material activated directly by inflammation (Ribeiro et al. 2020a). Studies show that this approach does not increase the material’s cytotoxicity or affect its mechanical properties but reduces its degradation rate (Ribeiro et al. 2022b).

Similarly, GelMA modified with metronidazole (MTR@gGel) has been synthesized. This material retained appropriate mechanical properties, did not hinder the differentiation of human dental pulp stem cells (hDPSCs), and exhibited robust antibacterial activity (Yan et al. 2021).

GelMA has also been combined with other antibiotics, including ciprofloxacin (Ribeiro et al. 2020b), azithromycin (Ayoub et al. 2022), minocycline, and clindamycin (Ribeiro et al. 2022a). Studies highlight the strong potential of this endodontic scaffold, capable of sustained antibiotic release while simultaneously driving tissue regeneration.

Furthermore, GelMA serves as an effective scaffold for stem cells, as confirmed by multiple studies. Electro-spinning has been used to fabricate GelMA loaded with hDPSCs (Yang et al. 2021) and stem cells from human exfoliated deciduous teeth (SHEDs) (Yuan et al. 2022). Cryopreservation of the material was feasible, and after thawing, the encapsulated cells maintained their ability to spread and proliferate *in vitro* (Yang et al. 2021). In a recent study, SHEDs were embedded in GelMA with simvastatin to stimulate odontoblastic differentiation, and the resulting material exhibited dual odontogenic and proangiogenic properties (Yuan et al. 2022).

Overall, GelMA hydrogels offer high biocompatibility, promoting cell adhesion, proliferation, and differentiation, which are essential features for pulp tissue regeneration (Sequeira et al. 2024). Besides bioactivity, GelMA is user-friendly due to its injectable form and on-demand photocuring ability. However, light-induced crosslinking generates free radicals that can damage cell membranes, leading to cell death. This drawback can be mitigated by optimizing photopolymerization parameters, such as reducing light intensity (Sequeira et al. 2024).

Despite this promising potential, extensive research is required before GelMA can be adopted for routine clinical use.

### Chitosan

Chitosan is a natural polysaccharide biopolymer obtained by the deacetylation of chitin, a structural component found in insect exoskeletons, crustacean shells, and fungal cell walls (Wu et al. 2019). It is characterized by inherent bacteriostatic activity, nontoxicity, and biocompatibility (Wu et al. 2019). Chitosan is widely investigated as a carrier for bioactive molecules in regenerative medicine and is used across the pharmaceutical industry as a drug delivery system in various formulations, including tablets, microspheres, hydrogels, and nanoparticles (Wu et al. 2019).

In dentistry, chitosan serves as an effective vehicle for agents such as chlorhexidine, calcium hydroxide, and triple antibiotic paste (Wu et al. 2019). This biopolymer is beneficial as it exhibits antibacterial activity, promotes the adhesion and differentiation of neural cells and DPSCs, enhances remineralization, and supports dentin regeneration in *in vivo* models. It also facilitates odontogenic differentiation, stimulates vascular endothelial growth factor (VEGF) secretion and cellular proliferation, and drives dentin-pulp complex regeneration (Wu et al. 2019).

Collagen-based systems, frequently reinforced with chitosan or crosslinked for structural stability, support pulp regeneration by mimicking the native extracellular matrix, thereby promoting angiogenesis and odontoblast differentiation (Desai et al. 2025). Chitosan–gelatin composites facilitate odontogenesis and mineralization (Siddiqui et al. 2022). Incorporating chitosan into fibrin hydrogels further enhances human dental pulp regeneration due to its antibacterial properties and minimal adverse effects on pulp cell morphology, viability, proliferation, and collagen matrix synthesis (Siddiqui et al. 2022).

Chitosan-based nanoparticles have attracted interest for their biocompatibility and capacity to deliver bioactive molecules involved in tissue regeneration. A recent systematic review of preclinical studies confirmed that chitosan-based nanoparticles enhance cell viability, odontogenic differentiation, angiogenesis, mineralization, and reparative dentin formation compared to conventional pulp-capping materials (Alqahtani et al. 2025). Consequently, chitosan-based nanoparticles and hydrogels represent highly promising scaffolds for VPT.

### Alginate

Alginate is an anionic linear polysaccharide derived from brown algae and composed of β-D-mannuronic and α-L-guluronic acid blocks (Farokhi et al. 2020). In VPT, it acts as a scaffold to support dentin and pulp regeneration. Alginate is widely used in regenerative dentistry due to its biocompatibility, biodegradability, nonimmunogenicity, and ability to form hydrogels through ionic crosslinking with divalent cations such as calcium (Chang et al. 2017; Sahoo and Biswal 2021). These hydrogels function both as a space-filling material – similar to the extracellular matrix with hydration properties promoting cellular wound healing – and as a carrier for cells and bioactive molecules (Abbass et al. 2020; Zhang et al. 2020). Both these properties are central to regenerative endodontics.

Alginate hydrogels can deliver exogenous growth factors or stimulate the release of endogenous growth factors from surrounding tissues, thereby enhancing natural dental pulp repair (Abbass et al. 2020). Various modifications of alginate are currently under research to enhance cell adhesion and proliferation, such as incorporating arginylglycylaspartic acid (RGD) peptides or laminin (Parmar et al. 2015). A recent work demonstrated that nanoclay-enhanced RGD-alginate microspheres delivered both DPSCs and VEGF and proved the potential of DPSCs-laden microspheres in *in vivo* pulp regeneration (Zhang et al. 2020). Furthermore, the addition of growth factors to stem cell cocultures within RGD–alginate scaffolds led to the creation of microenvironments that significantly enhanced the proliferation of combined DPSCs and human umbilical vein endothelial cells (Bhoj et al. 2015).

Despite these advantages, the standalone use of alginate in clinical applications is limited by its poor cell adhesion, low mechanical strength, and low degradability (Bhoj et al. 2015). To promote cell attachment, blending alginate hydrogels with gelatin has been widely investigated (Yu et al. 2019; Ferjaoui et al. 2024). This composite also serves as a bioink for 3D scaffold bioprinting, which allows precise control over scaffold interconnectivity and pore diameters to better mimic the native extracellular matrix (Yu et al. 2019).

Ultimately, alginate holds strong potential in VPT, particularly as a biocompatible and injectable scaffold that can deliver cells and growth factors to damaged pulp tissue. Chemical modifications or combining with bioactive materials significantly enhances its clinical viability in dental applications by addressing its innate drawbacks and expanding its regenerative capabilities.

### Platelet-rich fibrin

Platelet products have been utilized in TE and dentistry for over two decades as scaffolds for pulp regeneration (Fujioka-Kobayashi et al. 2020). PRF is obtained via centrifugation without anticoagulants, rendering it entirely autologous. Its fibrin matrix comprises platelets, leukocytes, and an array of cytokines and growth factors, including interleukin (IL)-1β, IL-4, IL-6, transforming growth factor (TGF)-β1, platelet-derived growth factor (PDGF), and VEGF (Lei et al. 2020). PRF also contains osteogenic cytokines and fiber holders, which act as a 3D centrifugal mesh capturing migrating cells and promotes sustained release of PDGF (Qian et al. 2017). This directly stimulates the proliferation and specialization of osteoblasts, endothelial cells, chondrocytes, and various types of fibroblasts (Rajasekar et al. 2025).

Clinical application of PRF in VPT is currently under research. A recent case report of two young patients with irreversible or chronic pulpitis detailed successful direct pulp capping (DPC) using PRF. Following caries removal and hemorrhage control, PRF was applied over the exposed pulp and sealed with MTA, glass ionomer cement, and composite resin. Both cases demonstrated pulp vitality, normal function, and an absence of pain or radiographic pathology at 6-month follow-up, indicating PRF may serve as a promising alternative to full endodontic therapy for certain cases of pulpitis (Bakshi et al. 2017).

Injectable PRF (i-PRF), an advanced liquid variant introduced in 2014, has higher regenerative potential (Liang et al. 2021). This scaffold offers enhanced flexibility and supports angiogenesis, modulates inflammation, and boosts bone regeneration and antimicrobial activity (Annamalai et al. 2018; Alia et al. 2023), making it a viable matrix for regenerative endodontic therapy (Huang et al. 2024). However, the application of PRF and i-PRF in VPT remains limited by a lack of standardized protocols (Liang et al. 2021).

Comparative research highlights the advantages of PRF over conventional materials used in VPT. In a clinical study, PRF demonstrated superior outcomes to calcium hydroxide and MTA in managing irreversible pulpitis, likely due to its natural scaffold properties and sustained release of growth factors (Singh et al. 2020). Furthermore, PRF showed high compatibility with human dental pulp cells, encouraging cell viability and mineralization, while remaining less cytotoxic and allowing more favorable pulp tissue viability than traditional capping materials (Dou et al. 2020; Panda et al. 2023). A randomized controlled trial confirmed that platelet concentrates are a promising alternative to MTA for DPC in adult permanent teeth with carious exposures, verified by clinical, radiographic, and cone-beam computed tomography observations of dentin bridge formation (Shobana et al. 2022). These findings position PRF as a biologically active and clinically effective option in VPT, offering regenerative advantages over conventional materials.

While PRF and its advanced form i-PRF show substantial promise compared to traditional alternatives like MTA due to their biological compatibility, regenerative properties, and adaptability, high-quality evidence, standardized protocols, and broader clinical validation are required to fully integrate them into mainstream endodontic practice.

### Demineralized dentin matrix

DDM is a biomaterial obtained from human or animal dentin through a process of defatting, demineralization, and sterilization. Demineralization exposes the organic components of dentin – primarily type I collagen – and matrix-bound growth factors (including bone morphogenetic protein-2, TGF-β, and VEGF), which are otherwise inaccessible in their mineralized state (Grawish et al. 2022). These processing steps render DDM highly bioactive (Liu et al. 2016).

The primary therapeutic benefits of DDM include osteoinduction and odontogenesis (stimulating the differentiation of odontoblast-like cells and osteoblasts) (Cunha et al. 2023), biocompatibility (well tolerated by tissues without inducing inflammatory responses) (Sultan et al. 2025), biodegradability (gradually decomposes, releasing growth factors at the site of application) (Holiel et al. 2023), and scaffold function (creates a natural framework promoting cell migration and adhesion) (Cunha et al. 2023).

Consequently, DDM is used in VPT in various forms. For example, DDM powder has been combined with gelatin and stabilized through photopolymerization. The resulting material was applied directly on the pulp as a capping agent. *In vitro* and *in vivo* studies showed that this hydrogel promoted pulp cell differentiation, supported mineralization, and was gradually biodegradable (Sedek et al. 2023).

Research also indicates that DDM extracts upregulate genes characteristic of odontoblasts (e.g., DSPP, DMP-1), leading to the formation of mineralized, dentin-like tissue in both *in vitro* (cell cultures) and *in vivo* (Cunha et al. 2023). Comparative studies reveal that DDM-based materials produce a thicker, more uniform dentin layer than calcium silicate-based materials like MTA (Sultan et al. 2025). Furthermore, DDM provides natural growth factors absent in silicate cements (Grawish et al. 2022) and can exhibit antibacterial and immunomodulatory effects when enriched with bioactive molecules (Xie et al. 2024).

Whether used for DPC or as a regenerative component following partial pulpotomy, DDM provides distinct biological advantages over traditional materials like MTA and Biodentine. Although further clinical studies are required, current evidence suggests DDM-based materials could become standard solutions in VPT to improve healing and natural tissue regeneration (Yi et al. 2024).

## Discussion

The natural scaffolds discussed here possess significant potential to improve regenerative pulp treatment. By providing structural support and a biologically favorable microenvironment, these biomaterials drive cell adhesion, proliferation, differentiation, angiogenesis, and tissue revascularization, ultimately leading to predictable pulp regeneration and improved long-term outcomes.

Despite these advantages, the clinical application of natural scaffolds in VPT remains challenging due to complex preparation, handling difficulties, a lack of standardized protocols, variable biological properties, and high treatment costs. Furthermore, because existing evidence is largely preclinical, well-designed clinical trials are necessary to confirm the safety, efficacy, and long-term performance of these materials.

Future research must prioritize optimizing scaffold composition, standardizing clinical procedures, and developing cost-effective strategies for routine implementation. Advancements in TE and regenerative dentistry position natural scaffolds as integral components of next-generation vital pulp therapies.

Based on the cited literature, a comparison of the reviewed biomaterials is presented in Table 2.

**Table 2:** Comparison of biomaterials used in vital pulp therapy (VPT) based on literature cited in this paper

Biomaterial	Key properties	Applications in VPT/endodontics	Limitations	Levels of evidence
GelMA	Biocompatible, light-curable hydrogel Modifiable with drugs (CHX, NaOCl, metronidazole, other antibiotics) Serve as a scaffold for stem cells	Drug and cell delivery Enzyme-activated, “on-demand” release (MMPs) Supports angiogenesis and odontogenesis	Requires precise control of photopolymerization (free radicals) Requires modification due to poor mechanical properties	*In vitro* *In vivo*
Chitosan	Antibacterial Bioresorbable Promotes DPSC differentiation Stimulates VEGF expression	Drug delivery (CHX, Ca(OH)_2_, tricalcium phosphate) Used in composites with collagen, gelatin, and hydroxyapatite Supports dentin–pulp regeneration	Data are mostly preclinical Requires extensive clinical studies	*In vitro* *In vivo*
Alginate	Biocompatible and biodegradable Forms hydrogels with Ca^2+^ ions Modifiable with RGD/laminin	Delivery of DPSCs and VEGF Used in microspheres and 3D structures Suitable for 3D printing of scaffolds	Poor cell adhesion Low mechanical strength Limited degradation control	*In vitro* *In vivo*
PRF/i-PRF	Autologous fibrin matrix with platelets, leukocytes, cytokines, and growth factors Supports angiogenesis, osteogenesis, and mineralization	Biological scaffold for pulp therapy Successfully used in pulpitis cases i-PRF offers better injectability and regenerative potential	Lack of standardized protocols Insufficient long-term clinical data	*In vitro* *In vivo* Clinical
DDM	Contains natural type I collagen and growth factors (BMP-2, TGF-β, VEGF) Bioactive, biocompatible, and biodegradable	Used for direct pulp capping Induces odontogenesis Yields better regenerative outcomes than MTA/Biodentine	Heterogeneity of formulations and lack of standardization Requires further clinical validation	*In vitro* *In vivo* Clinical

## Conclusions

Although natural biomaterials used in VPT exhibit promising bioactive and regenerative properties, their clinical utility remains limited. Preclinical and laboratory study results have not yet replaced conventional standards, such as MTA and Biodentine, which remain the benchmarks for conservative pulp treatment. However, the potential for biologically advanced solutions to preserve at-risk teeth is highly encouraging. Intensified research into TE applications in dentistry is essential to facilitate effective clinical integration and ensure patient access to modern, biologically driven treatments.

Due to the narrative nature of this review, no formal systematic protocol was applied, exposing the findings to selection bias, publication bias, and language bias, as only articles in English were considered. In addition, the choice of databases and search terms may have resulted in the omission of relevant studies. Nevertheless, utilizing multiple databases across diverse study types ensures a comprehensive overview of current knowledge regarding natural scaffolds in biological pulp treatment.
